# Inferring enterovirus D68 transmission dynamics from the genomic data of two 2022 North American outbreaks

**DOI:** 10.1038/s44298-024-00047-z

**Published:** 2024-08-02

**Authors:** Martin Grunnill, Alireza Eshaghi, Lambodhar Damodaran, Sandeep Nagra, Ali Gharouni, Thomas Braukmann, Shawn Clark, Adriana Peci, Sandra Isabel, Philip Banh, Louis du Plessis, Carmen Lia Murall, Caroline Colijn, Samira Mubareka, Maan Hasso, Justin Bahl, Heba H. Mostafa, Jonathan B. Gubbay, Samir N. Patel, Jianhong Wu, Venkata R. Duvvuri

**Affiliations:** 1 Public Health Ontario, Toronto, ON Canada; 2 Laboratory for Industrial and Applied Mathematics, Department of Mathematics and Statistics, York University, Toronto, ON Canada; 3 Department of Pathobiology, School of Veterinary Medicine, University of Pennsylvania, Philadelphia, PA USA; 4 Department of Laboratory Medicine and Pathobiology, Temerty Faculty of Medicine, University of Toronto, Toronto, ON Canada; 5 Axe Maladies infectieuses et immunitaires, Centre de recherche du CHU de Québec-Université Laval, Québec, QC Canada; 6 Department of Biosystems Science and Engineering, ETH Zürich, Basel, Switzerland; 7 Swiss Institute of Bioinformatics, Lausanne, Switzerland; 8 National Microbiology Laboratory, Public Health Agency of Canada, Montreal, QC Canada; 9 Department of Mathematics, Simon Fraser University, Burnaby, BC V5A 1S6 Canada; 10 Sunnybrook Research Institute, Toronto, ON Canada; 11 Center for the Ecology of Infectious Disease, Department of Infectious Diseases, Department of Epidemiology and Biostatistics, Institute of Bioinformatics, University of Georgia, Athens, GA USA; 12 Division of Medical Microbiology, Department of Pathology, Johns Hopkins School of Medicine, Baltimore, MD USA; 13 Department of Pathology and Laboratory Medicine, Faculty of Medicine, University of British Columbia, Vancouver, BC Canada

**Keywords:** Viral epidemiology, Viral evolution, Epidemiology

## Abstract

Enterovirus D68 (EV-D68) has emerged as a significant cause of acute respiratory illness in children globally, notably following its extensive outbreak in North America in 2014. A recent outbreak of EV-D68 was observed in Ontario, Canada, from August to October 2022. Our phylogenetic analysis revealed a notable genetic similarity between the Ontario outbreak and a concurrent outbreak in Maryland, USA. Utilizing Bayesian phylodynamic modeling on whole genome sequences (WGS) from both outbreaks, we determined the median peak time-varying reproduction number (R_t_) to be 2.70, 95% HPD (1.76, 4.08) in Ontario and 2.10, 95% HPD (1.41, 3.17) in Maryland. The R_t_ trends in Ontario closely matched those derived via EpiEstim using reported case numbers. Our study also provides new insights into the median infection duration of EV-D68, estimated at 7.94 days, 95% HPD (4.55, 12.8) in Ontario and 10.8 days, 95% HPD (5.85, 18.6) in Maryland, addressing the gap in the existing literature surrounding EV-D68’s infection period. We observed that the estimated Time since the Most Recent Common Ancestor (TMRCA) and the epidemic’s origin coincided with the easing of COVID-19 related social contact restrictions in both areas. This suggests that the relaxation of non-pharmaceutical interventions, initially implemented to control COVID-19, may have inadvertently facilitated the spread of EV-D68. These findings underscore the effectiveness of phylodynamic methods in public health, demonstrating their broad application from local to global scales and underscoring the critical role of pathogen genomic data in enhancing public health surveillance and outbreak characterization.

## Introduction

Enterovirus D68 (EV-D68) virus was first isolated in California, USA, in 1962^1^. In 2014, EV-D68 emerged as a prevalent cause of acute respiratory illness in children, receiving considerable attention due to its association with neurological syndromes such as acute flaccid myelitis (AFM) and transverse myelitis^2,3^. The epidemic behavior of EV-D68 viruses has exhibited a recurrent pattern of seasonality with epidemics following a biennial cycle^4,5^. North America experienced its most recent EV-D68 outbreaks between July and October 2022, which were linked to severe respiratory illnesses^6–9^.

EV-D68 belongs to species D of the *Enterovirus* genus of the *Picornaviridae* family^10^. This virus is a non-enveloped virus with a short (~7.5 kb) positive-sense single strand RNA genome, featuring a single open reading frame situated between two short flanking untranslated regions. EV-D68 exhibits considerable intra-type diversity due to its rapid antigenic evolution over time, resulting in three distinct clades: A, B and C^11,12^. Of these three clades, B was found to exhibit continuous evolution since 2014 which resulting in three sub-clades B1, B2 and B3. Phylogenetic analyses reported the presence of multiple globally proliferated sub-groups of B3 since 2016, and that B3 was responsible for more severe respiratory and neurological symptoms in pediatric populations^13–15^.

Phylodynamic methods use pathogen genetic sequences to construct phylogenies to study the diversification of pathogens at different spatio-temporal scales, while inferring key epidemic patterns of transmission between locations^16–18^. Hodcroft et al.^12^ reported key aspects of EV-D68 antigenic evolution, showing that age structure within populations has important implications for the diversification of surface proteins and host-specificity of lineages. Other phylodynamic analyses of EV-D68 have studied the relatedness between major epidemics and global circulation^11,19,20^.

Non-polio enteroviruses like EV-D68 are not nationally notifiable infections in North America. As such, case documentation is low which leads to a gap in our understanding of the transmission dynamics of these viruses. For instance, information on the infection period of EV-D68 is limited. Most enterovirus infections are found to shed from the upper respiratory tract over 1–3 weeks^21^. A previous epidemiological modeling study of EV-D68 used an infection period of 7 days^22^, which was derived from poliovirus^23,24^. Tambyah et al.^25^ described EV-D68 as having 1–5 days of incubation period and an infectious period from 1 day before to 5 days post symptom onset but give no reference. Mild symptoms of a median duration of 6 days (range 3–10 days) were observed during an EV-D68 outbreak at an elder care facility^26^.

In this study, we aim to address knowledge gaps on EV-D68’s transmission dynamics. We investigate the evolutionary history and relatedness of EV-D68 in Ontario and on a global scale, with a specific focus on the 2022 outbreak. We provide a contextual perspective by examining the outbreak’s geographical patterns within the B3 sub-clade of EV-D68 viruses. In particular, we find a high degree of genetic relatedness between samples from the 2022 outbreak, in Ontario, Canada^6^ and in Maryland, United States^7,9^. We investigate the epidemic transmission (via estimation of time-varying reproduction number, R_t_) through phylodynamic modeling of these two outbreaks, while shedding light on the infection duration of EV-D68 viruses. With EV-D68 reporting dates being available for the Ontario outbreak, we compare R_t_ as estimated via phylodynamic methods to more conventional methods (analyzing case incidence data with EpiEstim)^27^. We present epidemiological parameters derived from genome sequences, which can offer actionable information for public health practitioners.

## Methods

### Ethics statement

This project has received ethics review clearance from Public Health Ontario’s Ethics Review Board (ethics file number: 2023-016.01). The need for informed consent was waived by Public Health Ontario’s Ethics Review Board as per Article 5.5B of the Tri-Council Policy Statement: Ethical Conduct for Research Involving Humans (TCPS2), Canada’s national research ethics policy, which states that researchers are not required to seek participant consent for research that relies exclusively on the secondary use of non-identifiable information.

### Specimen collection and whole genome sequencing of 2022 EV-D68 in Ontario

Specimens submitted to Public Health Ontario Laboratory (PHOL) between July 31 and October 30, 2022, were collected from individuals with respiratory symptoms across various healthcare facilities in Ontario as part of routine care. EV-D68 was identified in 60.1% (*n* = 238) of randomly selected enterovirus-positive specimens (*n* = 396), with a predominant presence in nasal or nasopharyngeal samples. The highest number of EV-D68 positive test results was found among children less than 5 years of age. None of these cases presented with AFM. Additionally, whole genome sequencing was conducted on 36.5% (*n* = 87) of the randomly selected EV-D68 positive specimens^6^.

EV-D68 WGSs were retrieved from the National Center for Biotechnology Information (NCBI) from which a consensus sequence was created. The consensus sequence used for designing three primer pairs with an overlap of ~600 bp spanning the entire genome (Supplementary Table S1). Total RNA was extracted using the NucliSENS EMAG system following manufacturer’s instructions (bioMérieux Canada Inc, St-Laurent, Quebec, Canada) and reverse transcribed into cDNA using LunaScript® RT SuperMix Kit (cat# M3010, New England BioLabs, Ipswich, MA, USA). The synthesized cDNAs were used as templates for amplification of 3 long overlapping fragments along the genome. Each PCR reaction of 25 μl included the following: 5 μl of Q5 Hot Start buffer (New England Biolabs, Ipswich, MA), 0.5 μl of 10 mM dNTP, 0.5 μl of Q5 High-Fidelity DNA Polymerase, 1 μL of primer mix (10 μM), 2.5 μL of template DNA, and 15.5 μL of PCR grade water. The following thermal cycling conditions were used on an ABI SimpliAmp thermocycler: initial denaturation at 98 °C for 2 min, followed by 45 cycles at 98 °C for 10 seconds and 65 °C for 1 min and 72 °C for 5 min followed by a final extension at 72 °C for 5 min. The presence of each PCR product was confirmed by electrophoresis on 1% agarose gel. Equimolar amounts of each PCR product from the three reactions were pooled and cleaned with AMPure XP beads (0.5 ratio) for Illumina library preparation. Paired-end libraries for the MiniSeq platform were generated using Nextera XT DNA Library Prep Kit (Illumina) and subsequently purified using Agencourt AMPure XP beads (Beckman Coulter). The quality and size of prepared libraries were measured on the Agilent 4200 Tape Station using a High Sensitivity D1000 ScreenTape and reagent (HSD1000). Pooled normalized specimens, at a final concentration of 1.2 pM, were loaded onto a MiniSeq High Output Reagent Kit (300-cycles) and sequenced on an Illumina MiniSeq. For sequence analyses, FASTQ files were imported into CLC Genomics Workbench version 8.0.1 (CLC bio, Germantown, MD, USA). Reads were trimmed and mapped to the reference EV-D68 genome NY328 (GenBank: KP745766.1). Sequences were annotated using VAPiD v1.6.7^28^ prior to their submission to GenBank.

### Retrieving publicly available EV-D68 sequence data

To perform phylogenetic analyses, additional EV-D68 sequences were obtained from the NCBI virus database at the end of May 2023. For Whole Genome Sequences (WGS) the following filters were applied to remove sequences: without sample collection date, sequences <5000 bp in length and sequences where the proportion of nucleotides unassigned was over 0.05. Along with the WGS data produced from the Ontario 2022 outbreak (*n* = 87), a global dataset of 1134 EV-D68 WGSs was curated.

### Sequence analysis and phylogenetic construction

The Nextstrain Augur v22.0.2^29^ pipeline was used to align the WGS (*n* = 1134, length >5000 bp) data via MAFFT v7.505^30^, build a maximum likelihood (ML) phylogenetic tree via IQTree v2.0^31^, and refine the tree and infer node ancestry via TreeTime v0.10.1^32^. Auspice v2.37.1^29^ was used for visualization of Augur outputs. The curated 2022 outbreak WGS datasets from Ontario (ON-2022, *n* = 87) and Maryland (MD-2022, *n* = 74) were utilized in building phylodynamic models. TempEst v 1.5.1^33^ was employed to check that the temporal signals in ON-2022 and MD-2022 datasets are strong enough to allow phylodynamic analyses. Both ML trees derived from the ON-2022 and the MD-2022 datasets demonstrate a strong association between genetic distances and sampling dates.

### Genome-based epidemiological modeling using Bayesian phylodynamics

Bayesian phylodynamic analyses were performed on the curated ON-2022 (*n* = 87) and MD-2022 (*n* = 74) EV-D68 WGS datasets. These datasets were analyzed using BEAST v2.7.5^34^. Birth-Death Skyline Serial (BDSS using BDSKY v1.5.0^35^) models were fitted to each dataset separately. We used an HKY85 site substitution model with four gamma rate categories to estimate the evolutionary rate and an optimal relaxed molecular clock model^36^ that assumes heterogeneous substitution rates across phylogenetic branches, with an initial mean clock rate of 0.003^11^.

Considering the possible infection periods put forward for EV-D68^21–23,25,26^, we fitted all BDSS models using a prior for the infection period with a mean of 7 days and a wide standard deviation so as to cover 3–21 days (Supplementary Fig. S1). Birth-Death models do not estimate rate of reproductive maturation. Therefore, the model assumes patients immediately become infectious upon infection and remain infectious until being removed, i.e. there is no latent or exposed period^37^. The mean infection period (δ^-1^) was inverted to become the death rate or rate of becoming uninfectious (δ, also called the recovery rate) and converted to years (i.e. δ = 1/7 days = 52 year^−1^). To produce a gamma distributed prior^38^ for δ, we used formulas ${shape}=\frac{{{mean}}^{2}}{{variance}}=12.018$ and ${scale}=\frac{{variance}}{{mean}}=4.3269$ with a standard deviation of 15 year^−1^. All other parameter priors are listed in Supplementary Table S2.

Three additional independent runs were performed for each model. The performance of these independent runs was evaluated using Tracer v1.7.2^39^, checking the convergence of parameter, posterior, and likelihood values, along with screening individual runs ensuring effective sample sizes (ESS) > 200^40^ for all parameters. We repeated the analyses until we obtained at least three model runs meeting the above convergence criteria. Log and tree files were then combined using Log Combiner v2.7.5^40^. The bdskytools package in R (available at: https://github.com/laduplessis/bdskytools) was then used to produce skyplot figures of R_t_ and KDE plots of epidemic origin from the combined log files. The Python v3.10 package Seaborn v0.12.2 was used to produce box-violin plots of other parameter posterior distributions.

### Mathematical analysis of 2022 EV-D68 outbreak in Ontario using case counts data

The package EpiEstim v 2.2-4^27^ in R v4.1.2 was employed to calculate the time varying reproduction number (R_t_) from the time-series data of laboratory-confirmed positive cases of EV-D68 in Ontario during 2022. Due to the challenge in obtaining the serial interval for EV-D68, a mean serial interval of 3.7 days with a standard deviation (SD) of 2.6 days, as observed in the related pathogen EV-71^41^, was used for R_t_ estimation via EpiEstim^27^. Considering this a sensitivity analyses was performed on the EpiEstim analyses assuming a mean serial intervals of 2 and 7 days, but keeping the same SD.

## Results

### Phylogenetics of Ontario 2022 EV-D68 in a global epidemic context

**Fig. 1:**
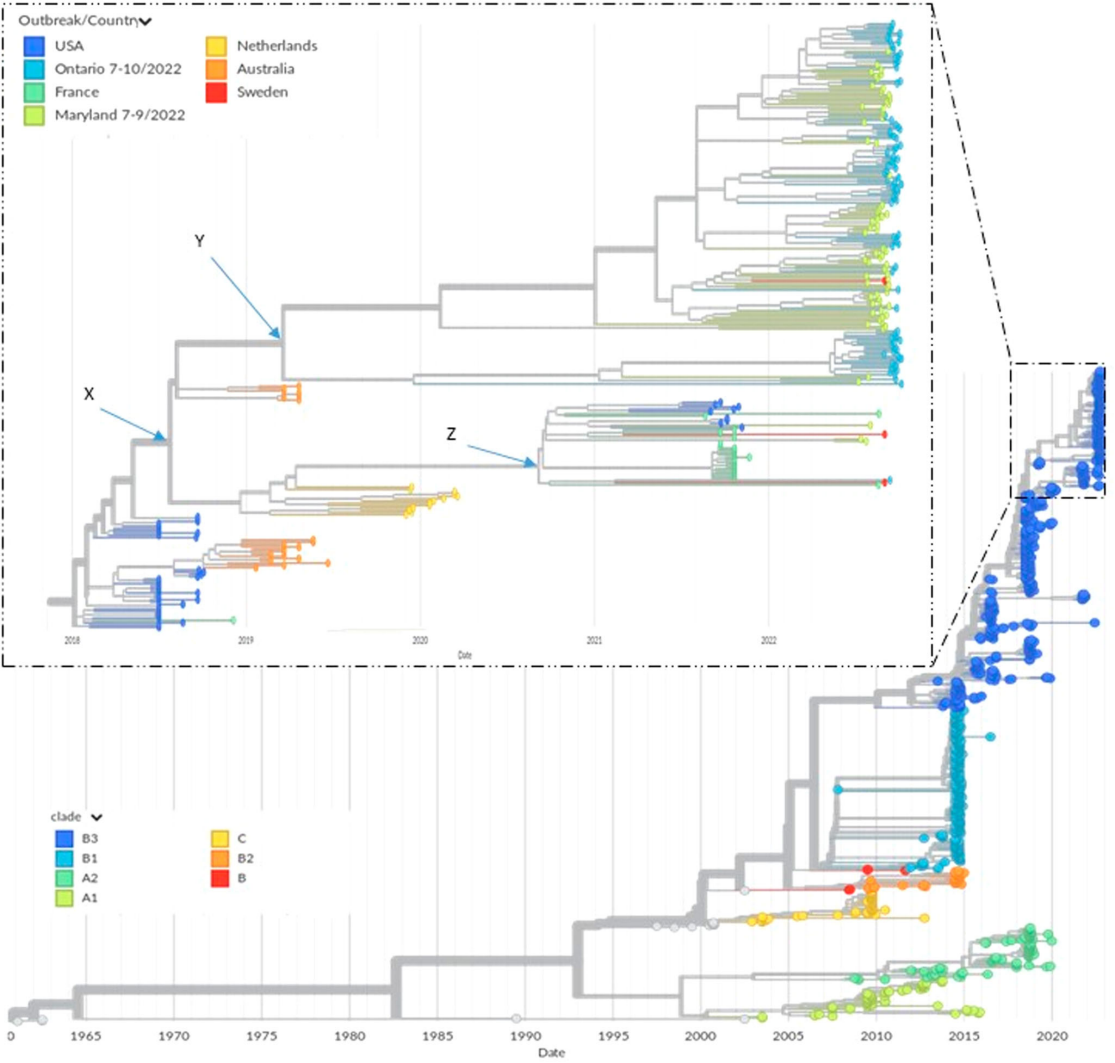
EV-D68 WGS based phylogenetic tree using Nextstrain^29^. The exert zooms on the 2022 EV-D68 outbreaks of Ontario, Canada and Maryland^7,9^. X, Y and Z are internal nodes of the tree referenced in the main text.

The Ontario 2022 EV-D68 isolates cluster with concurrent specimens from Maryland 2022^7^ along with a few concurrent sequences from Sweden and France (Fig. 1). All these isolates are of the B3 sub-clade and diversify from internal node **X** in Fig. 1, which is close to a cluster of US 2018 isolates. The majority of the Ontario 2022 isolates form a sub-grouping with isolates from the Maryland 2022 outbreak and a single Swedish 2022 isolate. This sub-grouping diversifies from internal node **Y** in Fig. 1, which is close to Australian 2019 (early) isolates. A single isolate from Ontario 2022, diversifying from the internal node **Z** as shown in Fig. 1, forms a sub-grouping with sequences from Maryland 2022, Sweden 2022, France 2021-2022 and other parts of the US in 2021. The edge leading to internal node **Z** clusters with other edges leading to isolates from a late 2019 to early 2020 outbreak in the Netherlands.

The amino acid (AA) alignment (Supplementary Fig. S2) of our ON-2022 VP1 sequences to the Fermon reference sequence (AY426531) revealed a total of 12 AA changes in the antigenic regions, specifically the BC loop (90–103, six AAs), DE loop (140–148, five AAs) and GH loop (209–219, one AA). A high degree of similarity was observed in these antigenic regions between ON-2022 and MD-2022^7^ outbreaks (Supplementary Figs. S3–S5). Similar to the Maryland outbreak, a combination of substitutions 95T (100%, 87/87) and 98A (36%, 23/87), in the BC loop was observed in the VP1 sequences from Ontario outbreak.

### Phylodynamic modeling and analysis: inference of evolutionary parameters

**Fig. 2:**
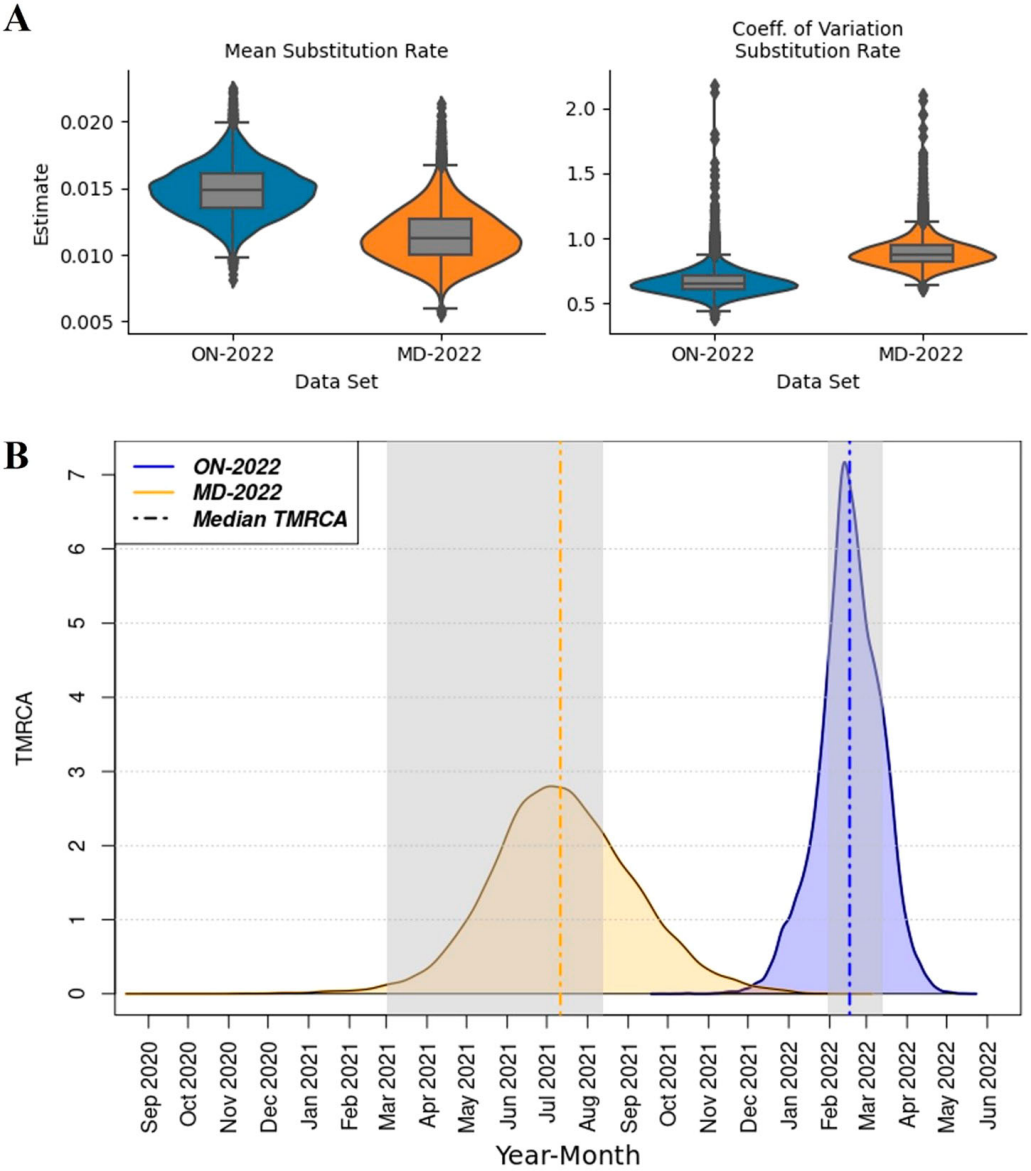
Distribution of posterior estimates of evolutionary parameters from 3 convergent runs of the best supported BDSS models fitted to different datasets of EV-D68 samples. **A** Box-Violin plots of posterior estimates of mean and the coefficient of variation for substitution rate (per site per year). **B** Kernel Density Estimate (KDE) plots of posterior estimates for TMRCA. The gray patches denote easing of COVID-19 restrictions in Maryland^55–57^ on the left and Ontario^54^ on the right. ON-2022 dataset contains all WGS sequences collected from Ontario 2022 EV-D68 cases. MD-2022 dataset contains WGS sequences collected from Maryland 2022 EV-D68 cases.

There are slight differences in the estimated median substitution rates between the BDSS models fitted to different datasets. The dataset ON-2022 has a median estimate of 0.0148 substitutions per site per year (95% Highest Posterior Density, HPD (0.0112, 0.0185) and the MD-2022 dataset a median estimate of 0.0113 substitutions per site per year with 95% HPD (0.00778, 0.0154). There is a higher coefficient of variation (median) of substitution rate for the MD-2022, 0.879, 95% HPD (0.706, 0.11) compared to ON-2022’s 0.655, 95% HPD (0.499, 0.838) (Fig. 2A), however the 95% HPD excludes 0 for both datasets, indicating strong support for a relaxed clock model^40^. The median Time since Most Recent Common Ancestor (TMRCA) estimate for the ON-2022 dataset was February 16, 2022, 95% HPD (December 26, 2021, March 31, 2022). For the MD-2022 dataset, the median TMRCA estimate was July 11, 2021, 95% HPD (April 3, 2021, November 3, 2021) (Fig. 2B).

### Phylodynamic modeling and analysis: inference of epidemiological parameters

**Fig. 3:**
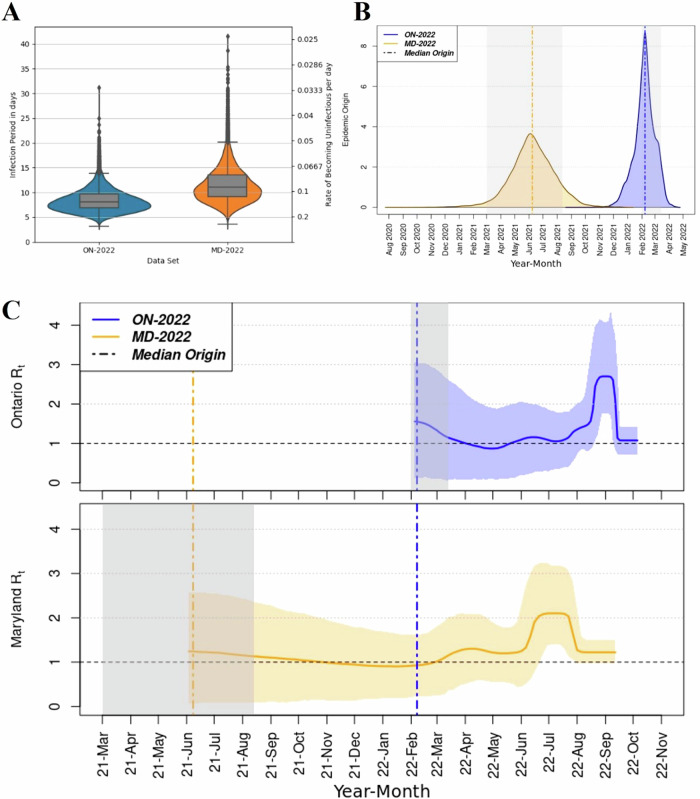
Distribution of posterior estimates of epidemiological parameters from 3 convergent runs of the best supported BDSS models fitted to different datasets of EV-D68 samples. **A** Box-Violin plots of posterior estimates of Infection period (Rates of Becoming non-infectious). **B** Kernel density estimate of estimated Epidemic Origin. **C top**: R_t_ estimates & 95% HPD intervals for ON-2022. **C bottom**: R_t_ estimates & 95% HPD intervals for MD-2022. The gray patches in **B** and **C** denote easing of COVID-19 restrictions in Maryland^55–57^ on the left and Ontario^54^ on the right. ON-2022 dataset contains all WGS sequences collected from Ontario 2022 EV-D68 cases. MD-2022 dataset contains WGS sequences collected from Maryland 2022 EV-D68 cases.

The estimated median duration of the infection period (in days) were 7.94 days, 95% HPD (4.55, 12.8) for the Ontario outbreak, and 10.8 days, 95% HPD (5.85, 18.6) for the Maryland outbreak (Fig. 3A).

The median epidemic origin estimate for the Ontario EV-D68 outbreak (dataset, ON-2022) was February 7, 2022, with a 95% HPD interval (December 21, 2021, April 20, 2022). In comparison, the Maryland EV-D68 outbreak (dataset, MD-2022) had a median epidemic origin estimate of June 8, 2021, with a 95% HPD (March 9, 2021, September 14, 2021) (Fig. 3B). Interestingly, the estimates of TMRCA for both epidemics overlapped with these epidemic origin estimates, as shown in Fig. 2A, B.

The BDSS models estimate a rise in the time-varying reproduction number (R_t_), leading to a plateaued peak occurring in the summer of 2022 (Fig. 3C). For the BDSS model fitted to the Ontario dataset (ON-2022), the R_t_ trend shows a higher and later, but shorter, peak compared to the model fitted to the Maryland dataset (MD-2022). The median R_t_ values for these peaks are 2.70, with a 95% HPD (1.76, 4.08) for the Ontario outbreak and 2.10, 95% HPD (1.41, 3.17) for the Maryland outbreak. Overall, the difference in R_t_ values suggests that the Ontario outbreak had a higher transmission rate at its peak compared to the Maryland outbreak, though the peak duration was shorter in Ontario.

### Mathematical analysis of 2022 EV-D68 outbreak in Ontario using case counts data

**Fig. 4:**
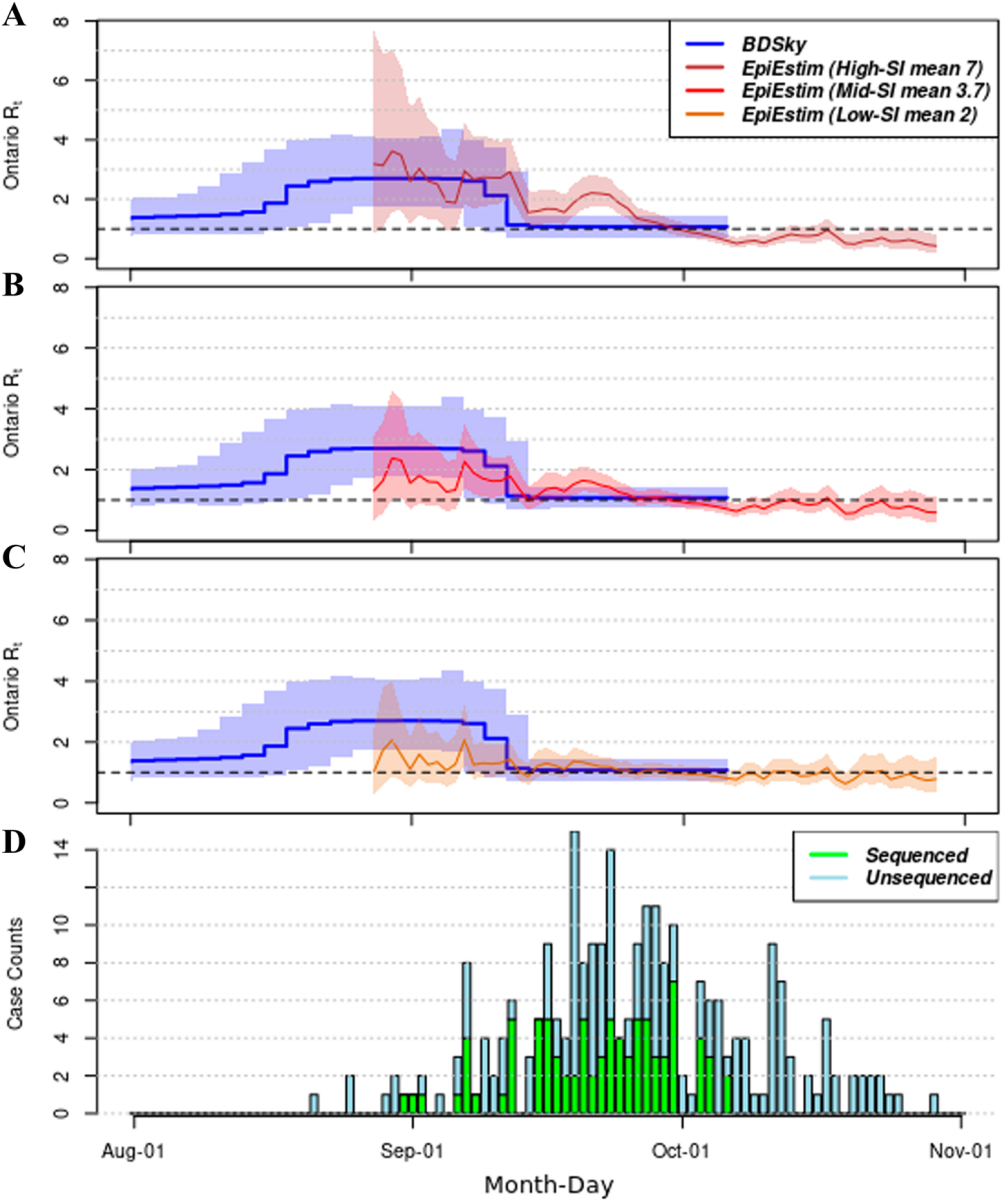
Comparison of time-varying reproduction number (R_t_) estimation from BDSKY (using genome sequence data) and EpiEstim (using case counts) methods, using Ontario 2022 EV-D68 data. Subplots **A**–**C**: Effective reproductive number estimated via three convergent runs of the best supported BDSS models fitted to the ON-2022 EV-D68 WGSs (blue) compared to estimation via EpiEstim case counts. All serial intervals (SI) used in EpiEstim method’s had an standard deviation (SD) of 2.6, The subplot **D** depicts Ontario EV-D68 case counts used in effective reproductive number estimation, only sequenced cases in green were used in the BDSKY based method. Note the BDSKY estimate of R_t_ goes back until February 2022 (Fig. 3C).

Figure 4 shows that R_t_ estimated via case counts has a comparable peak to the R_t_ estimates from the phylodynamic (BDSKY) analysis of genome sequence data. Likewise, both methodologies estimate a decline in R_t_ from early September 2022. R_t_ estimates produced by BDSKY and EpiEstim are more comparable when the higher serial interval (mean 7, SD = 2.6) was used for the EpiEstim based analyses. It should be noted that the case counts are a small samples size, and no genome sequence data were obtained from clinical cases beyond October 6, 2022 (Fig. 4D).

## Discussion

The phylogenetic and phylodynamic analyses reported here have demonstrated key features of two concurrent North American EV-D68 outbreaks and EV-D68’s epidemiology more widely. Through phylogenetic approaches we have demonstrated the epidemiological connection between the 2022 EV-D68 outbreaks in Maryland and Ontario. Our use of phylodynamic methods have aided in narrowing down the plausible window for EV-D68’s infection period. Furthermore, we have illustrated the practicality of phylodynamic methods in deriving R_t_ and epidemic origin. Notably, our epidemic origin estimates for the 2022 EV-D68 outbreaks coincide with the removal of restrictions aimed at curtailing the spread of COVID-19.

Cross-border disease dynamics between North American countries have been studied for other viral pathogen such as SARS-CoV-2, mumps virus and West Nile viruses^42–44^. In our analysis we demonstrate close genetic proximity between viruses circulating in Maryland and Ontario in 2022, where isolates captured from each outbreak cluster within the same sub-clade, B3, and share recent common ancestry. These viruses belong to the B3 clade of EV-D68 lineages which have previously been shown to play an important role in outbreaks in the region^13,45^. The combination of 95T and 98A substitutions in the BC loops correlates with increased mean viral load in clinical samples and a higher need for supplemental oxygen^7^.

Using phylodynamic modeling we were able to estimate important epidemiological parameters: the infection period and the time-varying reproduction number (R_t_). Our models showed highest support for a duration of infection of 7.94 days, 95% HPD (4.55, 12.8 days) for the ON-2022 and 10.8 days, 95% HPD (5.85, 18.6 days) for the MD-2022. Prior estimates of the infection period are lacking, however, respiratory viral shedding for enteroviruses has been documented to be between 1 and 3 weeks^21^. Specifically, the infection period for the EV-D68 ranges from 6 to 10 days^25^, and a symptomatic period from 3 to 10 days^26^. More recently Nguyen-Tran et al.^8^ found that the EV-D68 genome could be detected in the upper respiratory tract for a median of 12 days post symptom onset (7–15 days). Nguyen-Tran et al.^8^ point out that these RNA detection period should only be seen as an upper limit for infectious period. Given that infectious period (unlike infection period) does not include the latency period, Nguyen-Tran et al.’s^8^ findings are concurrent with our infection period of 7.94–10.8 days.

Our time-varying reproduction number (R_t_) estimates derived through phylodynamic methods produced similar values compared to deriving R_t_ through case count data and the serial interval (Fig. 4). Of particular note, greater concordance was seen when case count derived R_t_ values used a higher serial interval (mean = 7 days, SD = 2.6). Our mid-point serial interval for EV-D68 (mean = 3.6 days, SD = 2.6) was based on EV-71^41^, but given our estimate of the EV-D68 infection period (7.94–10.8 days) and an upper limit for the infectious period of 12 days^8^, EV-D68’s serial interval is likely to be closer to the higher value used in our sensitivity analysis. Previous epidemiological estimates of R_t_ from EV-D68 outbreaks (across several US states 2014-17) range between 0.5 and 1.6^22^. We find that our estimates of the median R_t_ (Fig. 4) were just over 1 in non-epidemic periods and 2.70 (95% HPD 1.76, 4.08) in Ontario and 2.10 (95% HPD 1.41, 3.17) in Maryland during the respective peak epidemic periods. A build up in the susceptible population due to reduced contacts over 2020–2021 may have led to the increased R_t_ values observed in Ontario and Maryland 2022, compared to estimates from several US states over 2014-17, a pre-pandemic period^22^. More generally, our EV-D68 BDSKY derived R_t_ estimates are consistent with other respiratory pathogens particularly other enteroviruses^24,46–49^. Park et al. ^22^ observed that increases in EV-D68 cases occurred later in the year for US states with more northern latitudes (2014–2019). Similarly, we find that in 2022, EV-D68 R_t_ values rose earlier in more southerly Maryland than Ontario.

The WGS-based substitution rates reported here, 0.0148 substitution per site per year (95% HPD 0.0112, 0.0185) for ON-2022 and 0.0113 substitution per site per year (95% HPD 0.0078, 0.0154) for MD-2022, are substantially higher than reported previously, 0.003 substitution per site per year^11^. The 38 WGS used in the analysis Eshaghi et al.^11^ come from 14 different countries, span 1960–2014 and therefore come from different EV-D68 clades, whereas, the WGS being used in our analyses are from one regional outbreak of a single sub-clade, B3. Time varying evolutionary metrics have been observed before, with faster rates observed when samples are drawn from shorter time periods^50–52^. Ghafari et al.^53^ demonstrated that during the SARS-CoV-2 and pH1N1 influenza pandemics this time varying evolutionary rate could be attributed to a short-term buildup of mildly deleterious mutations, that were eradicated over a longer term through purifying selection. This process of incomplete purifying selection may be the reason for the discrepancy between the EV-D68 substitution rates reported here and earlier^11^.

The estimated 2022 EV-D68 epidemic origin and TMRCA statistics from BDSS models for both regions coincide with the periods when measures to reduce social contact, known as Non-Pharmaceutical Interventions (NPIs), were relaxed. Specifically, in Ontario, Canada, the period was from January 31, 2022 to March 14, 2022, coinciding with the decline of the Omicron COVID-19 wave^54^. Meanwhile, the Maryland data corresponded with the phase of winding down several NPIs aimed at curtailing the spread of COVID-19, from early February 2021 to August 13, 2021^55–57^. NPIs aimed at controlling COVID-19 transmission have also significantly reduced influenza cases, virtually eliminated respiratory syncytial virus (RSV) hospitalization and diminish detectable circulation of several enteroviruses^58–61^. Therefore, it is possible that the coinciding of our epidemic origin and TMRCA estimates with the lifting of NPIs demonstrates the suppressing effect of NPIs on EV-D68 transmission. However, Fig. 1 depicts 2022 EV-D68 Maryland and Ontario sequences interspersed with each other and sequences from Sweden. This pattern suggests that the 2022 EV-D68 outbreaks in Ontario and Maryland may be the result of several independent introductions into their respective populations, and not a single introduction. This would mean that our R_t_ estimates are more likely to be for EV-D68 outbreaks in regions greater than Ontario or Maryland the further back in time the estimate is. Likewise, this may mean that our TMRCA and origin estimates are for EV-D68 outbreaks occurring over a much wider region than Ontario or Maryland.

This study has limitations that should be addressed in further research efforts. For instance, the above caveats over R_t_, TMRCA and origin estimates have, in part, come about through sampling in acute healthcare settings during an ongoing transmission within the wider community (Fig. 4C). It is important that sampling efforts are broader and capture more localities nationally, as well as broadly in North America, if not globally. As seen in the wider phylogenetic analysis (Fig. 1) there are long branches across the phylogeny which may indicate prolonged periods of within-host evolution, missed infections or un-sampled diversity. Thus, active surveillance is critical in identifying major source and sink populations for the EV-D68 virus, directing intervention efforts effectively. In addition to sampling biases, it is important that clinical observation studies of positive cases are conducted to validate the in-silico estimates of infection period for EV-D68 viruses to robustly model epidemiological dynamics further.

Future study of EV-D68 in a phylodynamic framework will not only be bolstered by wider sampling efforts but will also be aided by the inclusion of secondary metadata to study the importance of different host traits on viral evolution and diffusion. If metadata pertaining to severity of infection, age, and travel history of a patient is available phylodynamic methods can be used to determine the importance of traits in the diffusion process and potentially identify host characteristics that can inform control measures^62,63^. In summary, this study underscores the importance of pathogen genome surveillance combined with phylodynamics in complementing conventional epidemiological approaches within public health investigations.

## Supplementary information

Supplementary Materials

## Data Availability

All genome sequences of the 2022 Ontario EV-D68 outbreak isolates obtained in this study were submitted to GenBank under the accession numbers PP474817 to PP474903. The 2022 Maryland EV-D68 genome sequences were obtained from GenBank (accession numbers OP321139-OP321154, OP389245-OP389246, OP572035-OP572095)^7^.

## Code availability

The code developed in this study and the BEAUTi-created xml files are available on GitHub at Grunnill-Duvvuri-co-publications/Transmission-dynamics-inferred-from-Enterovirus-D68-genomic-data-from-2022-North-American-outbreaks (github.com).
