# Supplementary material for: Inferring enterovirus D68 transmission dynamics from the genomic data of two 2022 North American outbreaks
Source: Npj Viruses. 2024 Aug 2;2:34. doi: 10.1038/s44298-024-00047-z (PMC11721450; doi:10.1038/s44298-024-00047-z)
Supplement: Supplementary file 1 — Supplementary Materials [file 44298_2024_47_MOESM1_ESM.pdf]

Supplementary Material

Inferring Enterovirus D68 Transmission Dynamics from the Genomic Data of Two 2022 North American outbreaks

Martin Grunnill[1,2], Alireza Eshaghi[1], Lambodhar Damodaran[3], Sandeep Nagra[1], Ali Gharouni[1], Thomas Braukmann[1,4], Shawn Clark[1], Adriana Peci[1], Sandra Isabel[1,5], Philip Banh[1], Louis duPlessis[6,7], Carmen Lia Murall[8], Caroline Colijn[9], Samira Mubareka[4,10], Maan Hasso[1], Justin Bahl[11], Heba H. Mostafa[12], Jonathan B. Gubbay[1,13], Samir N. Patel[1,4], Jianhong Wu[2], and Venkata R. Duvvuri[1,2,4,†, *]

[1] Public Health Ontario, Toronto, Ontario, Canada

[2] Laboratory for Industrial and Applied Mathematics, Department of Mathematics and Statistics, York University, Toronto, Ontario, Canada

[3] Department of Pathobiology, School of Veterinary Medicine, University of Pennsylvania, Philadelphia, Pennsylvania, USA

[4] Department of Laboratory Medicine and Pathobiology, Temerty Faculty of Medicine, University of Toronto, Toronto, Ontario, Canada

[5] Axe Maladies infectieuses et immunitaires, Centre de recherche du CHU de Québec-Université Laval, Québec, QC

[6] Department of Biosystems Science and Engineering, ETH Zürich, Basel, Switzerland

[7] Swiss Institute of Bioinformatics, Lausanne, Switzerland

[8] National Microbiology Laboratory, Public Health Agency of Canada, Montreal, Canada

[9] Department of Mathematics, Simon Fraser University, Burnaby, BC V5A 1S6, Canada.

[10] Sunnybrook Research Institute, Toronto, Ontario, Canada

[11] Center for the Ecology of Infectious Disease, Department of Infectious Diseases, Department of Epidemiology and Biostatistics, Institute of Bioinformatics, University of Georgia, Athens, Georgia, USA

[12] Johns Hopkins School of Medicine, Department of Pathology, Division of Medical Microbiology, United States

[13] Department of Pathology and Laboratory Medicine, Faculty of Medicine, University of British Columbia, Vancouver, British Columbia, Canada

1. Methods

Table S1: Primers used to sequence whole EV-D68 genome.

PCR	primer	sequence (5'-3')	Position in genome*	Amplicon size (bp)
**1**	EV_D68-F1_For	TTAAAACAGCCTTGGGGTTG	1- 20	2764
	EV_D68-F1_Rev	ACAGTTGTGAGTATAGTGATCTCAGC	2739- 2764	
**2**	EV_D68-F2_For	ATAATACCTTGGATTAGTGGATCCCA	2145-2170	2736
	EV_D68-F2_Rev	CACAGATCAATGGGCAGCATCT	4860- 4881	
**3**	EV_D68-F3_For	AAAGTACGCACCACTTTACGCA	4283- 4304	3045
	EV_D68-F3_Rev	CCAAGTGGCCAAAATTTACC	7309- 7328	
***Relative position to Enterovirus D68 (EV-D68) isolate NY328 (GenBank: KP745766.1)**				

Table S2: Common Parameter Priors used in Birth-Death Skyline Serial Models

Parameter description	Distribution	Distribution Value 1	Distribution Value 2
Optimized lognormal relaxed clock rates	Lognormal (mean in real space)	Mean = 1.0	S.D. = 0.2
Optimized lognormal relaxed clock standard deviation.	Gamma	shape = 5	scale = 0.05
Optimized lognormal relaxed clock mean	Lognormal (mean in real space)	Mean = 0.003	S.D. = 1
Prior on gamma shape	Lognormal	Mean = 1.0	S.D. = 1.25
HKY transition-transversion	Lognormal	Mean = 1.0	S.D. = 1.25
Origin	Uniform	0.0	20.0
Reproduction number (R_e)	Lognormal	Mean = 0	S.D. = 1.0
Sampling proportion	Beta	α = 1	β = 9999

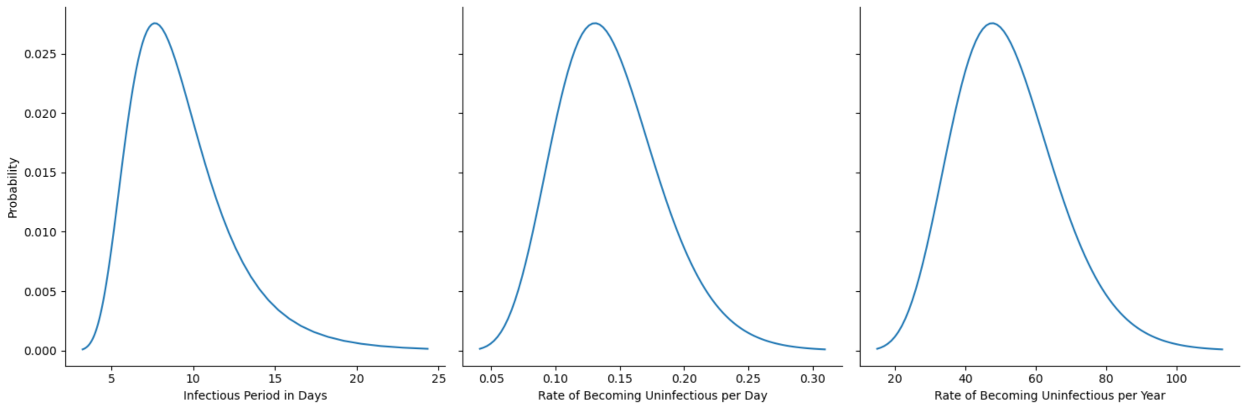

Figure S1: Prior used for Infection Period (δ^{-1}) or Rate of Becoming Uninfectious (δ).

The mean infection period (δ-1= 7 days) was inverted to become the rate of becoming uninfectious (δ, also called recovery rate) and converted to years (i.e. δ =1/7 days = 52 $year^{-1}$). To produce gamma distributed prior [43] for δ, we used formulas $\boldsymbol{shape = \frac{mean^2}{variance} = 12.018}$ and $\boldsymbol{scale = \frac{variance}{mean} = 4.3269}$ with a standard deviation of 15 $year^{-1}$.

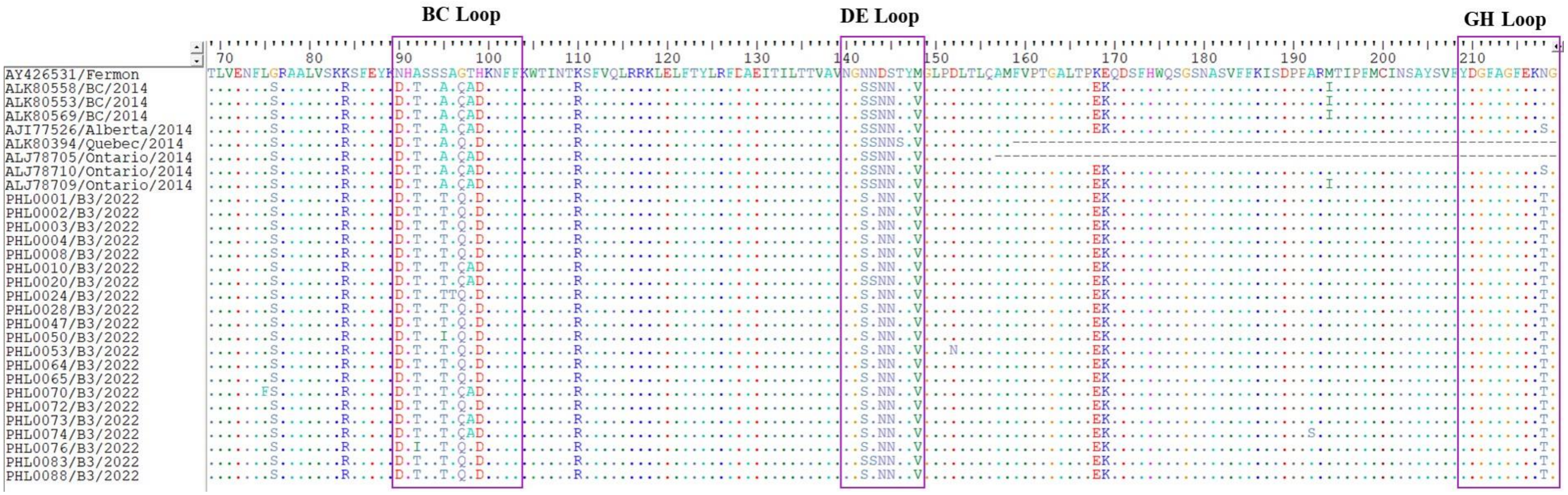

Figure S2. Amino Acid changes in the EV-D68 VP1 antigenic regions: BC loop, DE loop and GH loop.

BC: British Columbia

2. Results

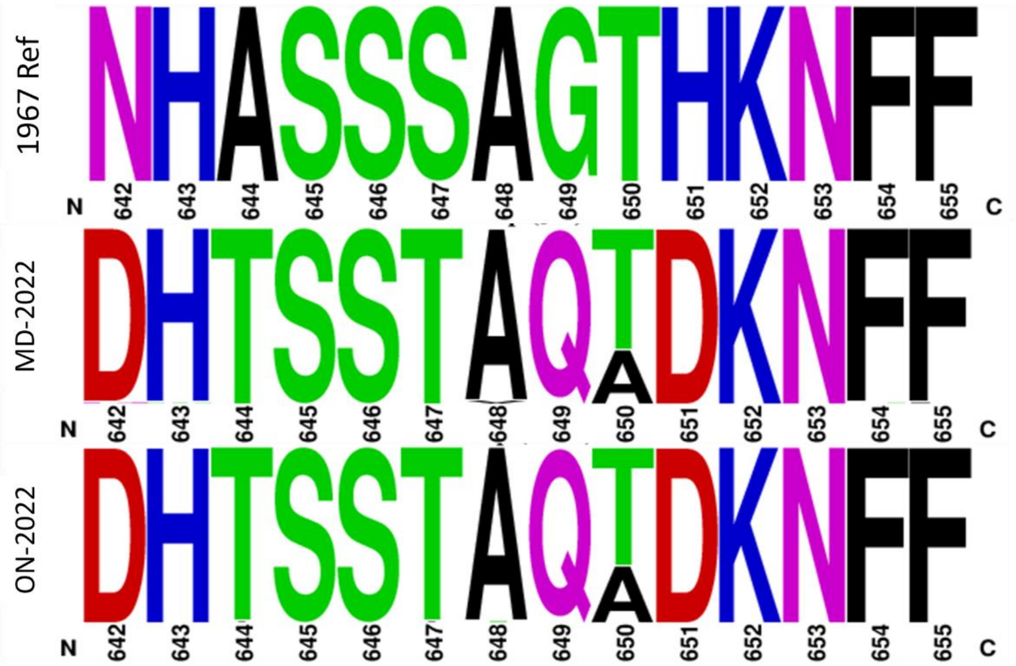

Figure S3: Amino acid logogram of VP1 BC loop.
1967 Fermon reference strain, AY426531 (top). MD-2022: Sequences from Maryland 2022 outbreak[1] (middle) and ON-2022: Sequences from Ontario 2022 outbreak (bottom). Logogram created using Crooks GE. *et al.* WebLogo - About (berkeley.edu)[2]. The numbering of the BC loop (642-655) in the EV-D68 polyportein corresponds to positions 90-103 in the VP1 protein.

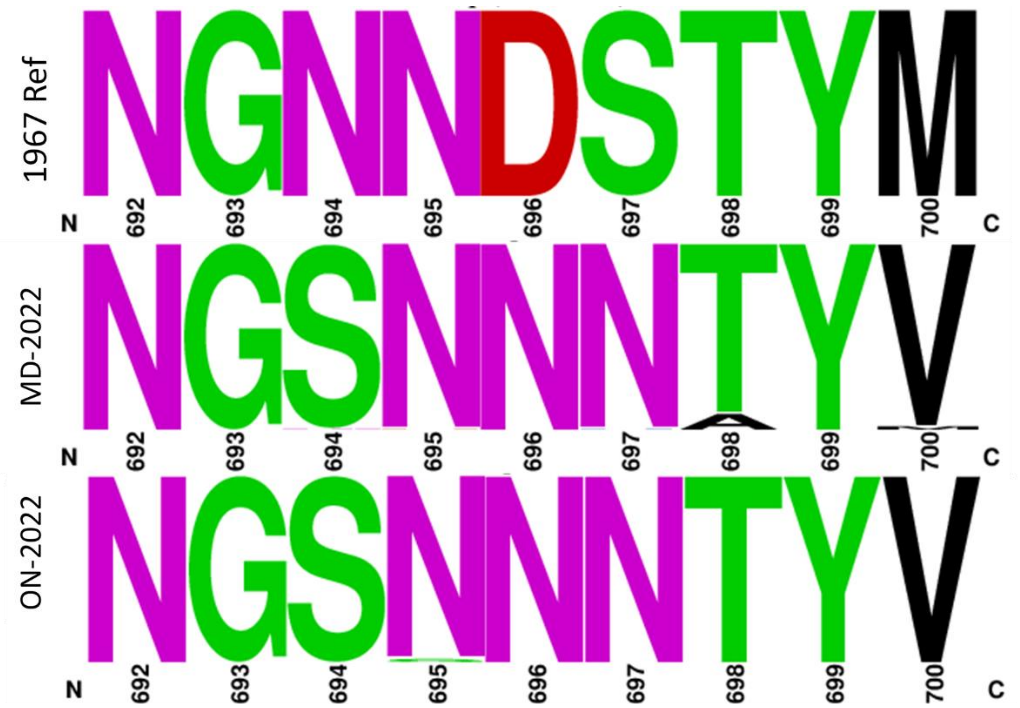

Figure S4: Amino acid logogram of VP1 DE loop.

1967 Fermon reference strain, AY426531 (top). MD-2022: Sequences from Maryland 2022 outbreak[1] (middle) and ON-2022: Sequences from Ontario 2022 outbreak (bottom). Logogram created using Crooks GE. *et al.* WebLogo - About (berkeley.edu)[2]. The numbering of the DE loop (692-700) in the EV-D68 polyportein corresponds to positions 140-148 in the VP1 protein.

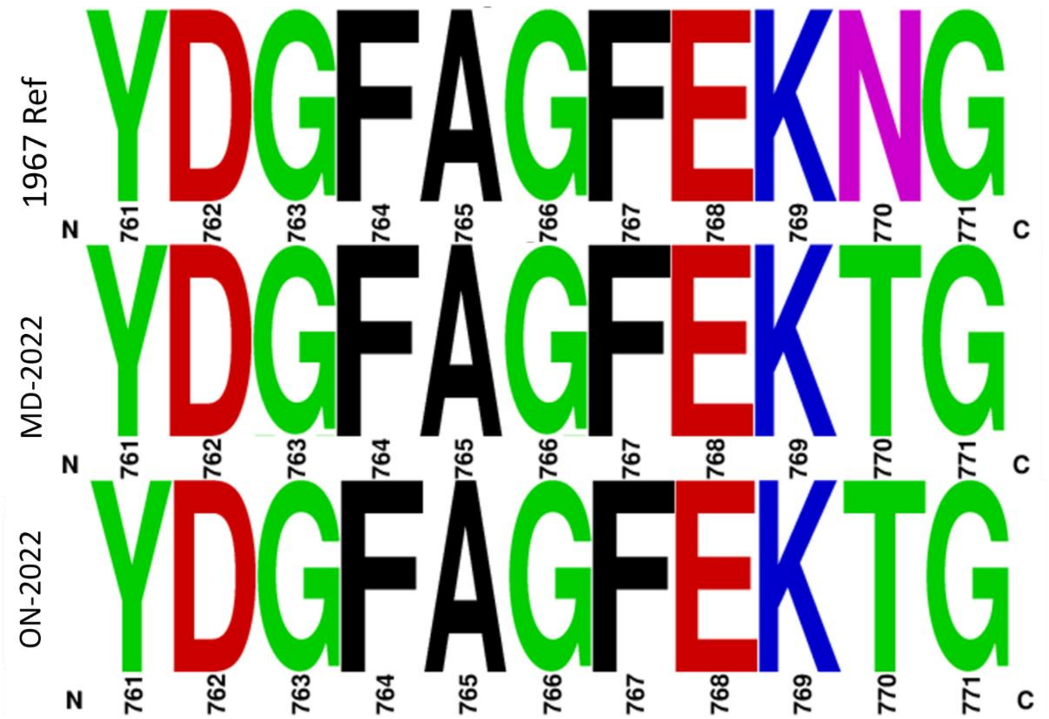

Figure S5: Amino acid logogram of VP1 GH loop.
1967 Fermon reference strain, AY426531 (top). MD-2022: Sequences from Maryland 2022 outbreak[1] (middle) and ON-2022: Sequences from Ontario 2022 outbreak (bottom). Logogram created using Crooks GE. *et al.* WebLogo - About (berkeley.edu)[2]. The GH loop polyportein-based numbering (761-771) corresponds to EV-D68 VP1 protein positions 209-219.

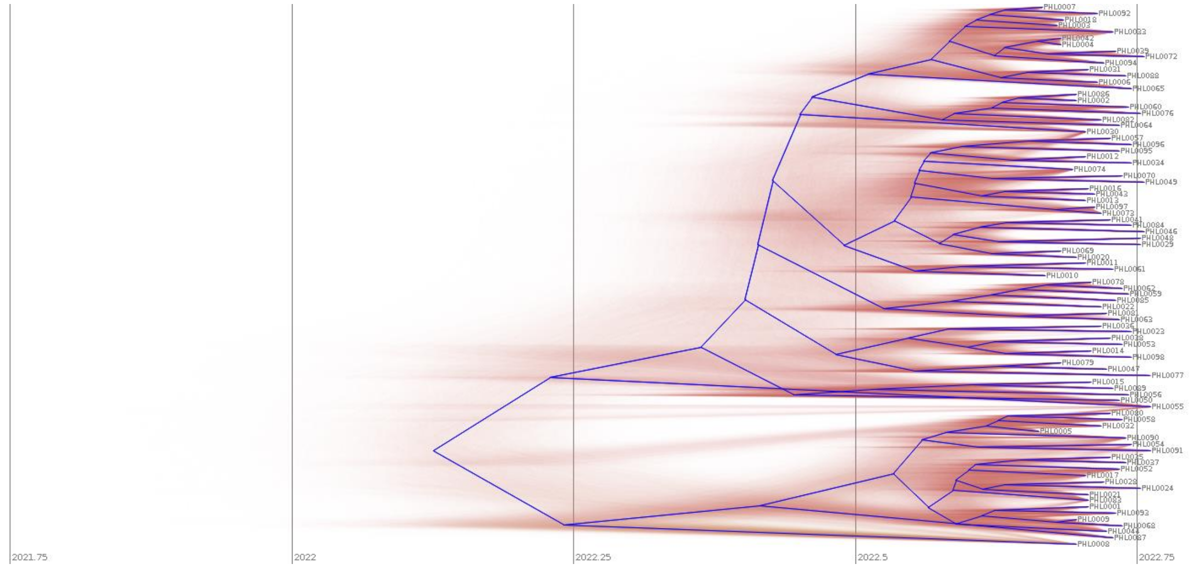

Figure S6: Summery tree plot of combined files from 3 runs of best performing BDSS model fitted to ON-2022 dataset. Plot produced using Densitree[3]. Summery root canal tree is in blue.

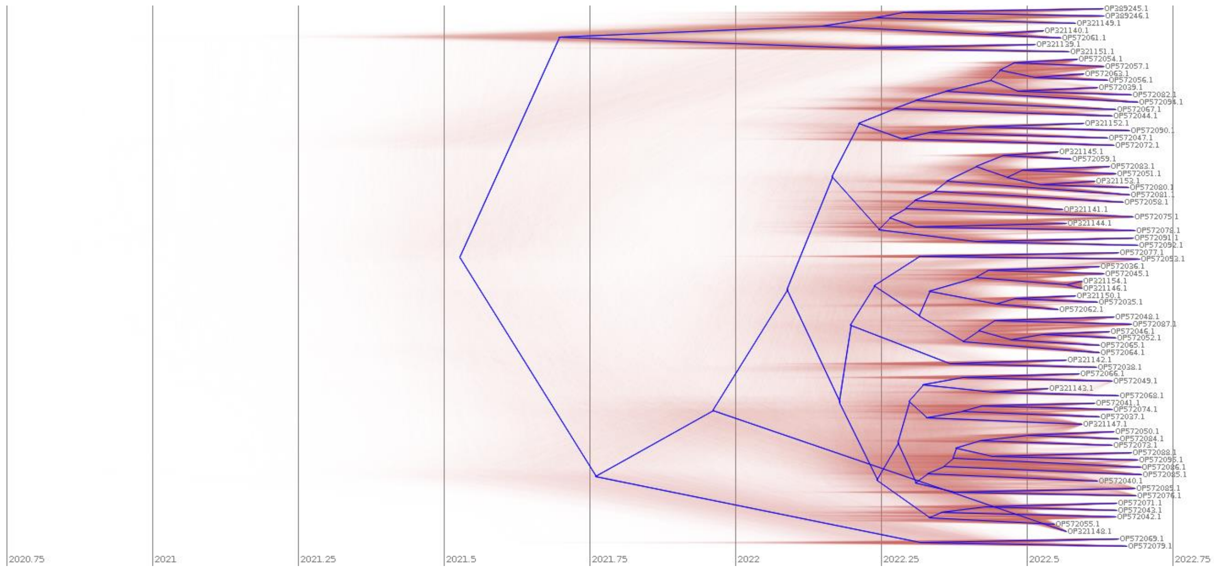

Figure S7: Summery tree plot of combined files from 3 runs of best performing BDSS model fitted to MD-2022 dataset. Plot produced using Densitree[3]. Summery root canal tree is in blue.

References

1. Crooks GE, Hon G, Chandonia JM, Brenner SE. WebLogo: a sequence logo generator. Genome Res. 2004 Jun;14(6):1188-90. doi: 10.1101/gr.849004.
2. Fall A, Han L, Abdullah O, Norton JM, Eldesouki RE, Forman M, Morris CP, Klein E, Mostafa HH. An increase in enterovirus D68 circulation and viral evolution during a period of increased influenza like illness, The Johns Hopkins Health System, USA, 2022. J Clin Virol. 2023 Mar;160:105379. doi: 10.1016/j.jcv.2023.105379.
3. Bouckaert RR. DensiTree: making sense of sets of phylogenetic trees. Bioinformatics. 2010 May 15;26(10):1372-3. doi: 10.1093/bioinformatics/btq110.
